# Effect of Dietary Inclusion of *Azadirachta indica* and *Moringa oleifera* Leaf Extracts on the Carcass Quality and Fatty Acid Composition of Lambs Fed High Forage Total Mixed Rations

**DOI:** 10.3390/ani12162039

**Published:** 2022-08-11

**Authors:** Edward Cottington Webb, Abubeker Hassen, Michael Olanrewaju Olaniyi, Pamela Pophiwa

**Affiliations:** Department of Animal Science, University of Pretoria, Hatfield, Pretoria 0028, South Africa

**Keywords:** bioactive compounds, methane mitigation, carotenoids, phytochemicals, medicinal plants, feed additives

## Abstract

**Simple Summary:**

Feed additives based on medicinal plants, such as neem and moringa plant extracts, are used to mitigate rumen methane emissions, but data regarding their effects on lamb meat quality are scarce. This study investigated the effects of oral supplementation of neem and moringa leaf extracts on the carcass quality and meat fatty acid composition of lambs. Neem leaf extracts had no effect on carcass fat and meat fatty acid composition. Whereas, Moringa leaf extract improved the meat fatty acid composition of lambs compared to the monensin treatment.

**Abstract:**

There is an increased interest in the use of medicinal plants as alternatives to antibiotic growth promoters and as agents for methane production mitigation. This study investigated the effects of *Azadirachta indica* and *Moringa oleifera* feed additives on the carcass and meat quality of lambs. Forty South African Mutton Merino lambs, weighing between 29 and 43 kg, were randomly assigned to four treatment groups (n = 10 lambs/treatment) and fed a basal total mixed ration (TMR) containing soybean meal (17%), yellow maize (28%), Alfalfa hay (20%), Eragrostis curvula hay (22.2%), molasses (6.0%), wheat offal (5%), urea (0.8%) and vitamin premix (0.5%) on a DM basis. The dietary treatments: TMR diet (control); TMR diet with *A. indica* leaf extract (*A. indica* leaf extract at a dosage of 50 mg per kg of feed: neem); TMR diet with *M. oleifera* leaf extract (*M. oleifera* leaf extract at a dosage of 50 mg per kg DM of feed: moringa); TMR diet with monensin (at a dosage of 50 mg monensin sodium per kg of feed: positive control). After an adaptation period of 10 days to the experimental conditions, the lambs from all treatment groups were fed ad libitum with the experimental diets. The lambs were slaughtered at a live weight of 60–65 kg after a 23 week trial period. The plant extract dietary additives had no significant effects on the carcass characteristics of the lambs. In comparison to monensin, supplementing with moringa leaf extracts resulted in a higher proportion of C18:1n9c (45.0% ± 0.57 vs. 40.5% ± 0.80; *p* < 0.05), total MUFAs (47.3% ± 0.66 vs. 42.6% ± 0.87; *p* < 0.05), and UFA:SFA ratio (1.01 ± 0.03 vs. 0.85 ± 0.03; *p* < 0.05), which may be beneficial for human health. Our results suggest that natural feed additives, such as *A. indica* and *M. oleifera* leaf extracts, can be included in lamb diets without compromising meat fatty acid composition. The negative economic impacts of such technologies on animal production and farm profitability should not be expected.

## 1. Introduction

There are increasing concerns regarding the long-term use of antibiotics in animal feed due to the fact of their potential impact on the environment and human health [1,2,3,4]. This fact has generated increased interest in research on the use of medicinal plants as a safe and inexpensive approach to replace the use of antibiotic growth promoters [5]. Multipurpose plants, such as *Azadirachta indica* and *Moringa oleifera*, are used in animal production for their medicinal and antioxidant properties and as possible mitigants for rumen methane emission [6]. The methane inhibitory effect of these plants is related to the presence of bioactive compounds, such as alkaloids, flavonoids and tannins, which are capable of interacting with rumen microbes and influencing ruminal fermentation patterns [6,7].

Although dietary inclusion of *A. indica* and *M. oleifera* and other plant extracts are promising strategies for mitigating rumen methane production [6], feeding strategies that alter rumen fermentation patterns may also affect lipid metabolism and, subsequently, meat quality [7]. Research has shown that phytogenic feed additives have an antimicrobial effect on bacterial species involved in rumen biohydrogenation, resulting in improved absorption and accumulation of polyunsaturated fatty acids (PUFAs) and conjugated linoleic acids (CLAs) in milk [8] and presumably meat. A previous study showed that supplementation with up to 5% neem fruit in lamb diets was effective in achieving high concentrations of rumenic acid (C18:2 cis-9 trans-11), a CLA which is beneficial for human health [9]. Feeding moringa silage (rich in α-linolenic acid) has been reported to increase both n-3 PUFA and CLA in lamb meat [10]. 

Most studies on *A. indica* and *M. oleifera* supplementation in ruminant diets focus on feeding whole plant parts, such as leaves, pods, fruits and seeds, to animals [9,10,11]. Plant extracts are often used in ethnoveterinary medicine and their inclusion as dietary additives is a useful strategy to conserve medicinal plants [12,13]. In vitro studies have shown the antimethanogenic properties of *A. indica* and *M. oleifera* plant extracts [14]; however, the effect of such antimethanogenic feed additives on animal production and meat quality has not been thoroughly examined. Farmers are less likely to adopt new technologies unless they are cost effective and induce no negative effects on animal production and product quality. Therefore, the objective of the current research was to investigate the effects of *A. indica* and *M. oleifera* plant extracts on the carcass traits and meat fatty acid composition of South African Mutton Merino (SAMM) lambs. 

## 2. Materials and Methods

### 2.1. Collection of Plant Materials and Extraction Procedure

The leaves from *A. indica* and *M. oleifera* trees were harvested in the South West region of Nigeria. The harvesting and handling of the plant materials from Nigeria to the University of Pretoria, South Africa, have previously been described [14]. On arrival, the leaves were freeze-dried for 5 days to a constant weight and milled through a 0.5 mm screen. The ground samples (100 g) were extracted with methanol (1 L) in glass vials and placed in a shaker for 96 h. The extracts were sieved through a 150 µm screen aperture, precipitated, freeze-dried to a constant weight and stored at 4 °C. 

### 2.2. Management of Experimental Animals

Forty weaned ram lambs (approximately 120–135 days old) with a mean body weight of 38.1 kg ± 3.83 were used in the present study in a completely randomised block design. The lambs were randomly allocated to four dietary treatment groups. Two lambs from each treatment within a block were allotted in a covered pen, with five pens of two lambs per treatment and a total of ten lambs per treatment. Each pen measured 3.2 × 2.2 m. The pens were considered experimental units, and the two sheep in each pen were the observational units. The lambs were kept at the Hatfield Experimental Farm of the University of Pretoria, in the city of Pretoria, South Africa. 

A total mixed ration (TMR) was formulated by a commercial feed company to meet the growth and maintenance requirements of the lambs. The TMR was sampled to conduct a chemical analysis. The dry matter (DM) and ash of the TMR used in this study were determined according to the standard procedure described by the Association of Official Analytical Chemists (AOAC) [15]. Acid detergent fibre (ADF), neutral detergent fibre (NDF) and lignin were determined using an Ankom technology 200/220 (Ankom Technology, Fairport, NY, USA) as described by Van Soest et al. [16]. Nitrogen was analysed using a Leco Instrumente GmbH, Kirchheim, Germany, nitrogen/protein analyser. The ether extract was determined by extracting the sample with ether following the Tecator Soxtec (HT6) system [15]. The formulation and chemical composition of the TMR is presented in Table 1.

All experimental animals received the same total mixed ration (TMR) during the experimental period. The TMR used was formulated to support an average daily gain (ADG) of approximately 250 g/head/day following the Agricultural Research Council’s [17] recommendations. The following four dietary additives were formed:

- TMR only: (control treatment);
- TMR plus *A. indica* leaf extract at a dosage of 50 mg per kg feed: (neem treatment);
- TMR plus *M. oleifera* leaf extract at a dosage of 50 mg per kg feed: (moringa treatment);
- TMR plus monensin sodium at a dosage of 50 mg per kg of feed: (monensin; positive control treatment).

The lambs from all treatment groups were fed ad libitum, and clean water was available ad libitum. Feed intake was calculated by subtracting the amount of refused feed from the feed offered the day before. A random sample was collected from the amount of feed offered each day as a retention sample for feed analysis later. The *A. indica* and *M. oleifera* extracts were reconstituted with distilled water (1 g extract to 1 L distilled water) and administered. The solutions of plant extract were administered at a dosage of 50 mL per kg of feed DM as recommended by Akanmu and Hassen [14]. The average DMI of the lambs was in the range of 1655 to 1866 g/head/day. The required dosages were drenched to lambs in the morning and afternoon before feeding using a 20 mL metal drencher (NJ Philips, Somersby, Australia). 

The initial body weights of the animals were recorded for three consecutive days before the start of the experiment and thereafter at seven-day intervals before the morning feeding until the end of the experimental period. The final weights of the animals were also recorded for three consecutive days before the morning feeding. The lambs were reared over a 23 week trial period to a marketable weight of 60–65 kg (9–10 months old). The data on feed intake, nutrient digestibility, average daily gain and methane measurement were document by Du Preez [18]. The lambs were slaughtered when they reached the required final weight of 48–52 kg, and the meat samples collected were used for the current study. 

### 2.3. Slaughter and Sampling Procedure

The lambs were slaughtered according to the standard procedure at the Renbro Abattoir, Hammanskraal, South Africa. Carcasses were immediately weighed to obtain the hot carcass weight and classified using the South African Carcass Classification System for beef, sheep and goat carcasses. This carcass classification system classifies carcasses based on their physical and compositional attributes, which include age (age categories: A, AB, B and C), carcass fatness (carcass fat codes: 1 to 6), carcass conformation (carcass conformation codes: 1 to 5) and damage (1 to 3) [19]. The carcasses were then chilled at 4 °C for 24 h. 

After 24 h in the chilling room, the carcasses were reweighed to obtain the cold carcass weight, and they were transferred to the laboratory for dissection under refrigerated conditions. Carcass composition was determined using the method described by Casey et al. [20]. Briefly, a three-rib sample was cut from the 8th, 9th and 10th lumbar vertebrae on the left side of each carcass, the ventral extremity of the sample being on a line drawn from the pubic symphysis to the middle of the first rib [20]. The three-rib cut sample was dissected into meat, fat and bone to obtain an estimate of the total carcass composition. The meat, fat and bone carcass components were vacuum packed and stored in the freezer at −20 °C until further analysis. Subcutaneous fat (SCF) and intramuscular fat (IMF) samples of approximately 5 g each were dissected from the three-rib-cut *Longissimus* muscle and stored in polythene bags at −20 °C for fatty acid analysis. 

### 2.4. Analytical Procedures

The dry matter content and ether extract of the *Longissimus* muscle samples were determined with the method used by the Association of Official Analytical Chemists [15]. The method involved boiling about 1 g of freeze-dried meat samples in petroleum ether for two hours and then oven drying until all the petroleum ether had evaporated. Thereafter, the samples were weighed and expressed as a percentage of the whole sample. Fat pigments were extracted according to the method of Kirton et al. [21]. The absorbance of each fraction was measured in a spectrophotometer (Specord 200^®^) at a 423 nm wavelength, and the lutein concentrations were calculated using Beer’s law equation [22].

### 2.5. Fatty Acid Analysis

The lipid extraction procedure and determination of fatty acid methyl esters were described by Webb and Casey [23]. Briefly, lipids were extracted in duplicate using a modification [24] of the chloroform:methanol (2:1, v/v) method [25]. Butylated hydroxytoluene (2.6 Di-tert-Butyl-p-Cresol) was included as an antioxidant. Methyl esters of the fatty acid component of the neutral triglycerides were prepared according to the NaOH/methanol method [15]. These esters were separated on a polar phase SP2330 column (2 m × 3 mm, packed with Silar 1OC coated on a Gas Chrom Q) fitted to a Shimadzu Tracera gas chromatograph with a barrier ionisation discharge detector. Profiles of the cis–trans fatty acids from the subcutaneous adipose tissues were obtained from fat samples that were treated with n-hexane at 35 °C for 24 h, after which the fatty acids were esterified according to the method of Van Wijngaarden [26]. The cis–trans fatty acids isomers were then separated on an SP2560 fused silica capillary column (100 m × 0.2 mm) fitted to a Varian 3700 gas chromatograph. Standards for the fatty acids were obtained from Nu-Chek-Prep., Inc. (Elysim, MI, USA). Fatty acids were expressed in both normalised (i.e., molar proportion) and gravimetric (i.e., milligrams per gram of fresh tissue) formats [27,28].

### 2.6. Statistical Methods

Data were analysed in a randomised complete block design. The variables of the carcass’ characteristics, lutein concentrations and meat fatty acids were first tested for normality and homoscedasticity with the Shapiro–Wilk and Levene’s tests, respectively. Statistical analysis was performed using the general linear model (GLM) ANOVA procedure in SPSS version 27, and the model included the treatment effect. Differences were considered significant at *p* < 0.05 and a tendency for significance at 0.05 < *p* < 0.10. The post hoc analyses were conducted with the Bonferroni comparison procedure in SPSS version 27. Factor component scores (z-scores) were calculated for the three-rib cut fat content to calculate the carcass fat content (CFC), after which the z-scores were transformed to standard scores (t-scores). Principal component factor analysis (PCA) was used to compute a succinct factor index for the carcass fat content (CFC t-score) and to describe the main effect of the feed additive treatments on the parameters measured. The measurements of the PCA plots were interpreted according to the correlations between each parameter. On the PCA plot, measurements close together are positively correlated; measurements separated by 180° are negatively correlated and measurements separated by 90° are independent [29]. 

## 3. Results

### 3.1. Carcass Characteristics

The results of the carcass characteristics of the lambs fed diets supplemented with neem (*A. indica)* and moringa (*M. oleifera)* leaf extracts are presented in Table 2. 

There were no significant differences in cold carcass weight among the dietary treatment groups. However, there was a tendency for a treatment effect on meat percentage (*p* = 0.06), fat percentage (*p* = 0.07) and CFC *T*-scores (*p* = 0.05) but the differences were not significant. Dietary treatment had no effect (*p* = 0.11) on dry matter and IMF content of the *Longissimus* muscle. 

### 3.2. Pigmentation of the Subcutaneous Fat

The lutein pigment concentration in the subcutaneous fat of the lambs supplemented with neem, moringa and monensin compared to the control are presented in Table 3. 

The content of the lutein pigment was affected by the dietary treatment (*p* = 0.03). The content of the lutein pigment was significantly higher (*p* < 0.05) in lambs fed with the control diet compared to those supplemented with neem or monensin (*p* < 0.05), while those fed with moringa had an intermediate (*p* > 0.05) lutein content. The practical implication is that the subcutaneous fat from the control (i.e., nonsupplemented) lambs was slightly more yellow compared to those supplemented with neem or monensin.

### 3.3. Fatty Acid Composition

The molar proportions of fatty acids in the SCF of lambs are presented in Table 4. The saturated fatty acids (SFAs) comprised 49.7–54.3% of the total fatty acids in the subcutaneous fat of the lambs. The main SFAs were palmitic acid (C16:0; 26–27.6%) and stearic acid (C18:0; 15.7–19.2%). There was a tendency toward a treatment effect on the proportion of stearic acid (*p* = 0.05). Although supplementation with the plant extracts did not affect the proportion of margaric acid (C17:0), docosanoic acid (C22:0) and tricosanoic acid (C23:0), monensin supplementation significantly decreased the C17:0 content and increased the proportion of both C22:0 and C23:0 compared to the control treatment (*p* < 0.05). 

The molar proportion of the monounsaturated fatty acids (MUFAs) accounted for approximately 45% of the total fatty acids. The major MUFA was oleic acid (C18:1n9c) which comprised approximately 43% of the total fatty acids and approximately 96% of MUFAs. The proportion of oleic acid (C18:1n9c) was affected (*p* = 0.02) by dietary treatment, since a significant difference was observed in the proportion of oleic acid (C18:1n9c) between the moringa and monensin treatment groups (45.0% vs. 40.5%). Similarly, total MUFAs were higher in the moringa treatment group compared to the monensin treatment group (47.3% vs. 42.6%). 

Total polyunsaturated fatty acids (PUFAs) accounted for approximately 2.8% of total fatty acids. Linoleic acid (C18:2n6c) was the main PUFA in the SCF of lambs in all groups. Dietary treatment affected total PUFAs (*p* < 0.01). Lambs supplemented with monensin had higher PUFAs in comparison to the control (2.53%; *p* < 0.05) and neem (2.56%; *p* < 0.05) treatment groups. This dietary treatment effect was the result of higher (*p* < 0.05) proportions of linoleic acid (C18:2n6c) and α-linoleic acid (C18:3n3) in the SCF of lambs supplemented with monensin (2.71%) compared to the control and neem treatment groups. 

Dietary treatment affected the UFA:SFA ratio (*p* = 0.03). Lambs in the monensin treatment group had lower (*p* < 0.05) UFA:SFA ratios compared to the control group and moringa treatment group. The n-6/n-3 (*p* = 0.78) and PUFA: SFA (*p* = 0.05) ratios were not affected by the dietary additives. 

### 3.4. Overview of the Feed Additive Treatment’s Main Effects on Physiological Parameters

**Figure 1:**
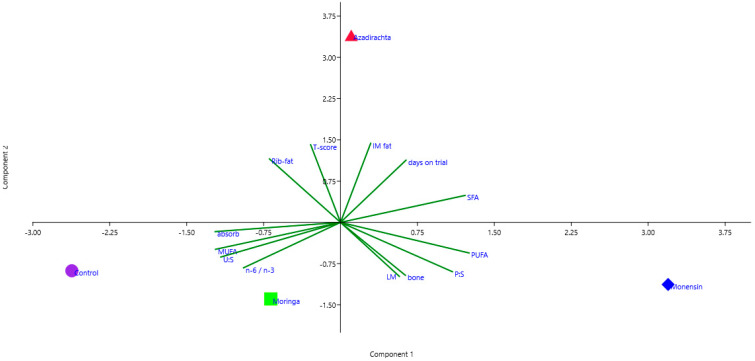
Projections of carcass and meat quality measurements on a plane defined by PC 1 (i.e., fatty acid composition component) and PC 2 (i.e., fat content component). Fat%: Rib-fat; carcass fat content t-scores: T-score); intramuscular fat%: IM fat; saturated fatty acids: SFA; PUFA:SFA—P:S; bone percentage: bone; meat%: LM; UFA:SFA—U:S; absorbance: absorb. Neem treatment: Azadirachta; monensin treatment: monensin; moringa treatment: moringa; negative control treatment: control.

The results of the PCA of the parameters considered in this study are presented in Figure 1. PC 1 (i.e., fatty acid composition component) is presented on the *x*-axis, and PC 2 (i.e., fat content component) is presented on the *y*-axis. The carcass fat content t-score, fat%, IMF% and days-on-trial showed a strong positive correlation with PC 2 (i.e., fat content component). Considering the direction of the PCA plot projections, it is evident that dietary supplementation with neem considerably increased the carcass fat content compared to the control and all of the other feed additive treatment groups. 

On the other hand, the proportions of PUFAs, SFAs and PUFA:SFA showed the largest positive correlations with PC 1 (i.e., fatty acid composition component), emphasising the improvements gained in terms of favourable fatty acids (i.e., MUFAs and UFA:SFA) by the supplementation of lambs’ diet with moringa and the control treatment compared to both the monensin and neem feed treatment groups. However, lambs supplemented with monensin showed an increase in PUFAs, PUFA:SFA, meat% and bone% more than the other treatment groups. 

## 4. Discussion

In the present study, lambs were fed to weights exceeding normal market weight (e.g., 60–65 kg) over a 23 week trial period to study the effects of plant supplements on carcass fat content and composition. The cold carcass weights were similar to those recorded in South African Mutton Merino (SAMM) lambs slaughtered at the same age/weight [30,31]. The meat percentage was lower, while the fat percentage was higher than previously reported for other South African lamb breeds in the A-age class [32] due to the differences in the slaughter age/weight. Future studies should consider slaughtering lambs when they reach a normal market weight, approximately 45 to 50 kg, for an ideal carcass quality that conforms to consumer preferences [30,33]. 

In terms of carcass composition, supplementing high-fibre diets with *A. indica* and *M. oleifera* leaf extract as antimethanogenic agents did not affect the meat and fat contents of the lamb carcasses. Previous studies have shown that the inclusion of *M. oleifera* in diets, shifts rumen fermentation kinetics from acetate to propionate, a major precursor of glucose synthesis in the liver that may be subsequently used for protein biosynthesis [34]. This effect is attributed to secondary bioactive compounds in *M. oleifera* plant extract, which have been reported to inhibit Gram-positive bacteria and favour propionate-producing bacteria [35]. The present results on carcass quality suggest that both *A. indica* and *M. oleifera* could be used in lamb diets as antimethanogenic additives, with the advantage of being natural additives that have no negative effects on carcass fatness. 

The degree of marbling is an important attribute of carcasses, and it is used as a visual cue by consumers to judge the quality of meat [36]. In the present study, the IMF values were within the range previously reported for lambs [37]. The IMF percentages of lambs were not significantly different across the four dietary treatment groups. The inclusion of *A. indica* and *M. oleifera* leaf extracts in lamb diets neither improved nor compromised the visual appearance of the meat. 

Yellow carcass fat is negatively evaluated by consumers in many countries [38]. Fat colour changes from a creamy white to a bright yellow–orange with the accumulation of carotenoids [22]. It is widely accepted that lutein is the main carotenoid in sheep adipose tissue [22,39,40]. Studies that quantify carotenoids in sheep fat are very scarce, because the concentrations are very low compared to carotenoids in cattle. The lutein concentrations found in the present study (0.57–1.16 mg/100 g feed) compare well to values previously reported in the literature for South African lamb breeds [33].

Previous studies have reported that plant secondary compounds have a protective effect on carotenoids resulting in higher depositions [41]. This effect was not observed in this study in both of our experimental treatment groups, which showed numerically lower lutein values compared to the control treatment. We can only speculate that low lutein concentrations in the neem and monensin treatment groups observed in this study were possibly related to their IMF content. Research has shown that as carotenoids accumulate in adipocytes, the increase in IMF may dilute the carotenoids and, consequently, reduce the yellowness of the subcutaneous fat [42]. More studies of the effect of medicinal plant extracts on the deposition of carotenoids in the adipose tissue of lambs/sheep should be considered.

Fat and long-chain fatty acids contribute to important aspects of meat quality and are key to the nutritional and sensory values of the meat [43]. In the present study, SFAs constituted approximately half of the total fatty acids in SCF of lambs, typical of SAMM lambs kept on high forage diets [23]. This is related to the fact that forages stimulate ruminal activity and biohydrogenation of fatty acids thus increasing the proportion of SFAs [44]. The main SFAs were palmitic acid (C16:0) and stearic acid (C18:0). This prevalence is in line with previously reported values for SAMM lambs kept on high forage-based diets [31].

Although SFAs are generally considered unhealthy, some have positive benefits on human health. It is only myristic acid (C14:0) and palmitic acid (C16:0) that are associated with an increased risk of obesity, hypercholesterolemia, some cancers and a decrease in LDL cholesterol [43,45]. Overall, our experimental diets did not alter the SFA proportion. The only SFAs affected by the dietary plant supplements were margaric acid (C17:0), docosanoic acid (C22.0) and tricosanoic acid (C23:0), and the difference was between the monensin and control treatment, while the moringa and neem treatments showed intermediate values. Our results indicate that the inclusion of *M. oleifera* and *A. indica* leaf extracts in lamb diets does not affect the SFA content of the meat and, by implication, does not cause increased risk to human health as previously suggested [46].

Oleic acid (C18:1n9c) was the main MUFA and conjugated linoleic acid (C18:2n6c) was the most prominent PUFA, as previously reported for lamb meat [45]. PUFAs (n-3 and n-6) are generally regarded as beneficial for human health [47]. However, high proportions of PUFAs can have negative effects on quality aspects such as fat firmness, shelf life and meat flavour [33]. In the present study higher proportions of oleic acid (C18:1n9c) were deposited in the SCF of lambs supplemented with moringa plant extract compared to monensin, which could be beneficial for human health. Higher proportions of linoleic acid (C18.2n6c) (n-6) were deposited in the SCF of lambs fed monensin compared to the neem and control treatment groups. Higher proportions and alpha-linoleic acid (C18:3n3) (n-3) were deposited in the SCF of lambs fed monensin-supplemented diets as opposed to other dietary treatment groups. However, the differences among dietary treatments in the proportions of PUFAs deposited in the SCF were minor (less than 1%) and will presumably not have any impact on human health, organoleptic properties and the technological quality of the meat.

The UFA:SFA ratio is commonly used to assess the nutritional value of fats, while the PUFA n-6-to-n-3 ratio indicates the risk of coronary heart disease or cancer in humans. In the present study, both the UFA:SFA and n-6/n-3 ratios were above the minimum recommended values of 0.4 and 4, respectively [43,47]. The differences across dietary treatments were not significant and, hence, were presumably of minor importance.

## 5. Conclusions

Despite small differences, the inclusion of *M. oleifera*, as a feed additive, resulted in higher oleic acid, MUFAs and UFA:SFA ratio compared to the monensin treatment, which could be considered beneficial for human health. *A. indica* had no or minimal effects on the carcass’ characteristics and the meat fatty acid composition. Overall, the antimethanogenic feed additives investigated in this study had no negative effects on the carcass fat and fatty acid composition of the lambs. Therefore, *A. indica* and *M. oleifera* feed additives can be used as safe and inexpensive antimethanogenic agents, without compromising the resultant meat quality.

## Figures and Tables

**Table 1:** Formulation and chemical composition on a DM basis of the total mixed ration.

Ingredient	Composition (%)
Yellow maize	28.0
Eragrostis curvula hay	22.2
Alfalfa hay	20.0
Soybean meal	17.0
Molasses	6.0
Wheat	5.0
Urea	0.8
Vitamin premix	0.5
*Parameter*	*Chemical composition* (%)
Dry matter	89.7
CP	17.2
Ash	6.5
Starch	6.5
NDF	3.4
ADF	24.2
Lignin	2.5
ME	0.9

**Table 2:** The effects of dietary inclusion of neem (*Azadirachta indica)* and moringa (*Moringa oleifera)* leaf extract on the carcass characteristics (LS means ± SE) of South African Mutton Merino lambs.

Treatment	Control	Neem	Moringa	Monensin	*p*-Value
Initial weight (kg)	38.6 ± 0.95	38.3 ± 1.29	38.1 ± 1.17	37.4 ± 1.43	0.95
CCW (kg)	29.2 ± 0.55	30.2 ± 0.65	30.8 ± 1.31	28.7 ± 0.92	0.61
Meat (%)	51.8 ± 1.11	51.6 ± 0.81	55.5 ± 0.86	54.4 ± 0.70	0.06
Fat (%)	33.2 ± 1.22	34.3 ± 0.77	30.1 ± 1.23	30.0 ± 0.85	0.07
Bone (%)	15.0 ± 0.40	14.1 ± 0.72	14.4 ± 0.61	15.6 ± 0.48	0.51
LM dry matter (%)	32.7 ± 0.63	34.5 ± 0.90	34.5 ± 1.70	35.8 ± 2.44	0.11
IMF (%)	12.0 ± 0.89	15.2 ± 0.71	12.2 ± 1.33	12.6 ± 0.97	0.28
CFC t-scores	50.3± 2.43	58.9 ± 2.15	45.4 ± 4.11	46.2 ±2.55	0.05

CCW: cold carcass weight; LM: longissimus muscle; IMF: intramuscular fat; CFC: carcass fat content.

**Table 3:** The effect of dietary inclusion of neem (*Azadirachta indica)* and moringa *(Moringa oleifera)* on the lutein concentrations in the subcutaneous fat of SA Mutton Merino lambs.

	Control	Neem	Moringa	Monensin	*p*-Value
Lutein (mg/100 g)	1.16 ^a^ ± 0.14	0.59 ^b^ ± 0.13	0.86 ^ab^ ± 0.14	0.57 ^b^ ± 0.14	0.03

^ab^ Means with different superscripts were significantly different (*p* < 0.05).

**Table 4:** The effects of dietary inclusion of *Azadirachta indica* and *Moringa oleifera* leaf extract on the fatty acid composition of the subcutaneous fat (w/w%; LS mean ± SE) of South African Mutton Merino lambs.

Fatty Acids	Control	Neem	Moringa	Monensin	*p*-Value
C14:0	3.09 ± 0.28	3.20 ± 0.22	2.76 ± 0.15	3.45 ± 0.24	0.43
C16:0	27.0 ± 0.66	27.6 ± 0.64	26.0 ± 0.82	26.6 ± 0.45	0.59
C17:0	5.52 ^a^ ± 0.44	3.74 ^ab^ ± 0.33	4.52 ^ab^ ± 0.15	3.59 ^b^ ± 0.44	0.03
C18:0	16.7 ± 1.31	17.6 ± 0.65	15.7 ± 0.64	19.2 ± 1.39	0.05
C20:0	0.11 ± 0.01	0.11 ± 0.01	0.11 ± 0.01	0.13 ± 0.01	0.37
C21:0	0.51 ± 0.03	0.41 ± 0.03	0.47 ± 0.05	0.53 ± 0.08	0.65
C22:0	0.02 ^a^ ± 0.00	0.03 ^ab^ ± 0.00	0.03 ^ab^ ± 0.00	0.04 ^b^ ± 0.00	0.04
C23:0	0.05 ^a^ ± 0.00	0.07 ^ab^ ± 0.01	0.06 ^ab^ ± 0.01	0.09 ^b^ ± 0.01	0.04
SFA	50.1 ^a^ ±1.01	52.8 ^b^ ± 0.55	49.7 ^a^ ± 0.69	54.3 ^b^ ± 1.16	0.03
C14:1	0.25 ± 0.03	0.17 ± 0.02	0.18 ± 0.01	0.15 ± 0.02	0.13
C15:1	0.03 ± 0.01	0.02 ± 0.01	0.03 ± 0.01	0.03 ± 0.01	0.85
C16:1	1.32 ± 0.13	1.07 ± 0.09	1.08 ± 0.08	0.96 ± 0.06	0.23
C18:1n9c	44.4 ^ab^ ± 1.05	42.4 ^ab^ ± 0.76	45.0 ^a^ ± 0.57	40.5 ^b^ ± 0.80	0.02
C18:1n9t	0.79 ± 0.15	0.59 ± 0.07	0.79 ± 0.17	0.95 ± 0.20	0.65
C20:1	0.15 ^a^ ± 0.01	0.08 ^b^ ± 0.00	0.14 ^ab^ ± 0.02	0.09 ^ab^ ± 0.01	0.02
MUFAs	46.9 ^ab^ ± 1.02	44.1 ^ab^ ± 0.69	47.3 ^a^ ± 0.66	42.6 ^b^ ± 0.87	0.02
C20:2	0.08 ± 0.00	0.07 ± 0.00	0.08 ± 0.00	0.08 ± 0.01	0.76
C18:2n6c	2.05 ^a^ ± 0.04	2.07 ^a^ ± 0.15	2.16 ^ab^ ± 0.11	2.71 ^b^ ± 0.11	0.01
C18:3n3	0.36 ^a^ ± 0.01	0.38 ^a^ ± 0.02	0.39 ^a^ ± 0.02	0.51 ^b^ ± 0.03	<0.01
C18:3n6	0.03 ± 0.00	0.03 ± 0.00	0.03 ± 0.00	0.04 ± 0.00	0.10
C20:3n6	0.03 ± 0.00	0.03 ± 0.00	0.03 ± 0.00	0.03 ± 0.00	0.38
PUFAs	2.53 ^a^ ± 0.06	2.56 ^a^ ± 0.18	2.66 ^ab^ ± 0.14	3.36 ^b^ ± 0.13	<0.01
UFA/SFA	0.99 ^a^ ± 0.04	0.89 ^ab^ ± 0.02	1.01 ^a^ ± 0.03	0.85 ^b^ ± 0.03	0.03
PUFA n-6/n-3	5.86 ± 0.13	5.47 ± 0.23	5.61 ± 0.15	5.56 ± 0.31	0.78
PUFA/SFA	0.05 ^a^ ± 0.00	0.05 ^a^ ± 0.00	0.05 ^a^ ± 0.00	0.06 ^b^ ± 0.00	0.05

^ab^ Means with different superscripts were significantly different (*p* < 0.05). SFAs: saturated fatty acids; MUFAs: monounsaturated fatty acids; PUFAs: polyunsaturated fatty acids.

## Data Availability

Data supporting the reported results are available upon request.
